# *Liberibacter*, A Preemptive Bacterium: Apoptotic Response Repression in the Host Gut at the Early Infection to Facilitate Its Acquisition and Transmission

**DOI:** 10.3389/fmicb.2020.589509

**Published:** 2020-12-23

**Authors:** Xiao-Tian Tang, Kelsy Fortuna, Azucena Mendoza Herrera, Cecilia Tamborindeguy

**Affiliations:** Department of Entomology, Texas A&M University, College Station, TX, United States

**Keywords:** *Candidatus* Liberibacter solanacearum, *Bactericera cockerelli*, gut, apoptosis, survivin, acquisition, transmission

## Abstract

“*Candidatus* Liberibacter solanacearum” (Lso) is a phloem-limited Gram-negative bacterium that infects crops worldwide. In North America, two haplotypes of Lso (LsoA and LsoB) are transmitted by the potato psyllid, *Bactericera cockerelli* (Šulc), in a circulative and persistent manner. Both haplotypes cause damaging plant diseases (e.g., zebra chip in potatoes). The psyllid gut is the first organ Lso encounters and could be a barrier for its transmission. However, little is known about the psyllid gut immune responses triggered upon Lso infection. In this study, we focused on the apoptotic response in the gut of adult potato psyllids at the early stage of Lso infection. We found that there was no evidence of apoptosis induced in the gut of the adult potato psyllids upon infection with either Lso haplotype based on microscopic observations. However, the expression of the inhibitor of apoptosis IAPP5.2 gene (survivin-like) was significantly upregulated during the period that Lso translocated into the gut cells. Interestingly, silencing of IAPP5.2 gene significantly upregulated the expression of two effector caspases and induced apoptosis in the psyllid gut cells. Moreover, RNA interference (RNAi) of IAPP5.2 significantly decreased the Lso titer in the gut of adult psyllids and reduced their transmission efficiency. Taken together, these observations suggest that Lso might repress the apoptotic response in the psyllid guts by inducing the anti-apoptotic gene IAPP5.2 at an early stage of the infection, which may favor Lso acquisition in the gut cells and facilitate its transmission by potato psyllid.

## Introduction

Apoptosis, one of the programmed cell death forms, is an essential physiological process that can occur in response to intracellular or extracellular signals. It plays a critical role in a variety of biological events including development and tissue homeostasis (Zimmermann et al., 2001; Liu et al., 2008). Apoptosis is an evolutionarily conserved mechanism orchestrated by multiple proteins (Kornbluth and White, 2005). Some of the key proteins mediating apoptosis are caspases, a family of conserved intracellular aspartate-specific cysteine proteases (Lamkanfi et al., 2002; Shi, 2004). Once an initiator caspase is activated, it processes downstream effector caspases that are responsible for apoptosis (Earnshaw et al., 1999). Apoptosis can be divided mainly into extrinsic or intrinsic pathway. In mammals, the extrinsic pathway is mediated by caspase-8, while the intrinsic pathway can be initiated through caspase-9. Both pathways trigger apoptosis through the cleavage of the downstream executioner proteins, caspases 3 and 7 (Elmore, 2007). Because apoptosis signaling mediated by caspases is an irreversible process, caspase activities must be precisely regulated in order to prevent the undesired death of the organism’s cells (Goyal, 2001; Liu et al., 2008). Direct inhibition of caspase activity by inhibitor of apoptosis proteins (IAPs) is one of the most important mechanisms for apoptosis inhibition (Bergmann, 2010). To date, eight human IAPs have been identified, including NAIP, c-IAP1, c-IAP2, XIAP, Survivin, Bruce, ILP-2, and Livin (Salvesen and Duckett, 2002). However, the *Drosophila* genome encodes four IAPs: DIAP1, DIAP2, dBruce, and Deterin, the latter protein is a survivin homolog (Hay, 2000; Xu et al., 2009). IAPs are characterized by the presence of one to three baculoviral IAP repeat (BIR) domains, which are required for binding and suppression of specific cell death-inducing caspases (Silke and Meier, 2013).

Apoptosis also plays essential roles in the innate immune system, leading to the rapid destruction of cellular structures or organelles (Danial and Korsmeyer, 2004; Green, 2005). Host cells can employ apoptosis as a defense mechanism to impede the replication and spread of intracellular pathogens. However, because intracellular pathogens often rely on the host cell machinery to complete their life cycle and also require intact host cells to shield themselves from the host immune defense system, many intracellular pathogens are able to manipulate the apoptosis pathways of the host cells (Gao and Abu Kwaik, 2000). Indeed, some pathogens such as *Shigella* and *Salmonella* can hijack the immune responses of the host and take control over the fate of the host cells (Gao and Abu Kwaik, 2000). These pathogens are either directly or indirectly engaged in the host cell apoptotic pathways, and many of them target the caspase signaling to impede apoptosis of the infected host cells (Liu et al., 2008).

“*Candidatus* Liberibacter solanacearum” (Lso) is a Gram-negative, intracellular and unculturable bacterium infecting crops worldwide. Presently, several Lso haplotypes (LsoA, LsoB, LsoC, LsoD, LsoE, LsoF, and LsoU) of this pathogen exist in the world (Glynn et al., 2012; Lin et al., 2012; Nelson et al., 2013; Haapalainen et al., 2018; Grimm and Garczynski, 2019), which are transmitted by several psyllid species and result in large yield loss among different crops. Haplotypes LsoA and LsoB are mainly present in North America where they are transmitted by the potato psyllid (also known as the tomato psyllid), *Bactericera cockerelli* (Šulc; Hemiptera: Triozidae). LsoA and LsoB can infect numerous solanaceous crops and cause damaging diseases such as zebra chip in potatoes (Liefting et al., 2009; Tamborindeguy et al., 2017). Lso is transmitted by psyllids in a circulative and persistent manner (Cooper et al., 2014; Cicero et al., 2016, 2017). Despite our understanding of its transmission route within the insect body, the molecular mechanisms underlying the transmission process remain largely unknown. The psyllid gut is the first organ Lso encounters and therefore provides an essential link for understanding the biology of Lso acquisition or transmission within the potato psyllid.

Importantly, the psyllid gut could act as a barrier for Lso transmission and determine Lso transmission efficiency. Indeed, the ability of the bacteria to infect the gut depends on the insect immune responses as well as the bacterial strategies deployed to disrupt the host immunity (Vyas et al., 2015). An increasing number of studies have demonstrated that apoptosis in insect vectors can be induced by insect-borne plant pathogens. For instance, apoptosis was observed in the gut of Asian citrus psyllid (*Diaphorina citri*) adults from the “*Ca*. *L. asiaticus*” (*C*Las)-infected colonies, but not in the nymphal gut (Ghanim et al., 2016; Mann et al., 2018). It is probable that the apoptotic response serves to limit the acquisition or transmission efficiency of *C*Las by the Asian citrus psyllid. Indeed, *C*Las titer increased at a faster rate when the bacterium was acquired by nymphs compared to adults (Ammar et al., 2016). In contrast to *C*Las, no evidence of apoptosis was uncovered in the gut of the Lso-infected adult potato psyllids in our previous study (Tang and Tamborindeguy, 2019). However, all these studies focused on insects from infected colonies, which most likely acquired the bacteria during the nymphal stages. It is still possible that apoptosis is induced at the early stages of infection, but as the infection becomes persistent, as in the case of adults from the Lso-infected colonies, there is no evidence of apoptosis. Another possibility is that Lso could affect the gut immune response to favor its acquisition or transmission. Interestingly, while adults can efficiently transmit LsoA and LsoB if the pathogens were acquired during the nymphal stages, we have discovered that adults can acquire and transmit LsoA with lower efficiency than LsoB (Tang et al., 2020b). Therefore, it is still necessary to investigate the apoptotic response in the gut of psyllids upon Lso infection.

In the present study, we investigated the apoptotic response in the potato psyllid gut during the early stages of Lso infection. First, the accumulation of the two Lso haplotypes A and B was determined during the early infection period. Second, the occurrence of changes in nuclear morphology, actin cytoskeleton, and the integrity of the gut cell DNA were evaluated because these changes are hallmarks of apoptosis. Third, the expression of apoptosis-related genes (Tang and Tamborindeguy, 2019) was evaluated at key time points during the early infection period. Here, we showed that apoptosis is not induced in the gut of the adult potato psyllids upon infection of either Lso haplotype; however, one of the inhibitors of apoptosis, IAPP5.2 gene (survivin-like), was significantly activated. Silencing of IAPP5.2 significantly decreased the Lso titer in the gut of adult psyllids and reduced its transmission efficiency. These observations suggest that Lso could be exploiting the psyllid’s cell machinery to avoid the apoptotic immune defenses by utilizing an inhibitor of apoptosis, thereby facilitating its acquisition and transmission. This study not only provides insights into the interactions occurring between Lso and its insect vector, the potato psyllid, at the gut interface, but could also represent a stepping stone toward the development of novel control strategies to disrupt the pathogen transmission within insect vector.

## Materials and Methods

### Insect Colonies and Tomato Plants

Lso-free, LsoA‐ and LsoB-infected psyllid colonies were maintained separately on tomato plants (Moneymaker; Victory Seed Company, Molalla, OR) in insect cages (24 × 13.5 × 13.5 cm, BioQuip®, Compton, CA) at room temperature 24 ± 1°C and photoperiod of 16: 8 h (L: D) as described in Yao et al. (2016).

To obtain Lso-infected tomato plants, 6-week-old tomato plants were infected as described in Nachappa et al. (2014) using three male psyllids harboring LsoA or LsoB, respectively. After 1 week, the psyllids were removed from the tomato plants. Around 3 weeks after Lso inoculation, the plants were tested for Lso infection using the LsoF/OI2 primers (Li et al., 2009) and the Lso haplotype in the plants was confirmed using the Lso SSR-1 primers (Lin et al., 2012).

### Psyllid Exposure to Lso and Gut Dissection

**Figure 1:**
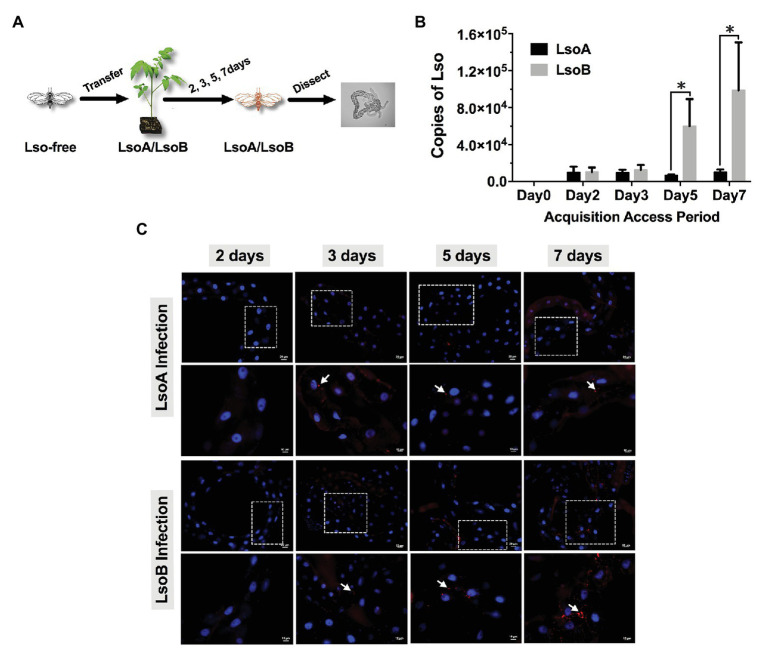
Quantification and immunolocalization of *Candidatus* Liberibacter solanacearum (Lso) in the gut of potato psyllids following acquisition. **(A)** Adult psyllids were transferred to LsoA‐ or LsoB-infected tomato plants for 2-, 3-, 5-, and 7-day acquisition access periods (AAPs) and then their guts were dissected for quantification and immunolocalization as shown in the schematic representation. **(B)** Quantification analysis of Lso copies in the gut of potato psyllids following Lso acquisition. The bars represent the copies of LsoA (black) and LsoB (gray) in pools of 50 guts following a 0-, 2-, 3-, 5-, and 7-day AAP. Data represent means ± SD of three independent experiments. * indicate statistical differences at *p* < 0.05 using the Student’s *t* test. **(C)** Immunolocalization of LsoA and LsoB in the gut of potato psyllids following Lso acquisition. The white dashed rectangle indicates the enlargement region of upper panels. White arrows indicated the Lso signals. The Lso signals can be first observed at infection day 3. The bars of upper and lower panels are 20 and 10 μm, respectively. Psyllid and tomato plant diagrams were made by Dr. Ordom Huot. The gut picture is from Tang et al. (2019).

Approximately 7-day-old Lso-free adult psyllids were transferred to LsoA‐ or LsoB-infected tomato plants. To determine the acquisition profile of LsoA and LsoB in the gut of psyllids, insects were collected after 2-, 3-, 5-, or 7-day acquisition access periods (AAPs) on LsoA‐ or LsoB-infected plants (Figure 1A). For each specified exposure time, the guts from the LsoA‐ or LsoB-exposed psyllids were dissected under the dissecting microscope as described in Ibanez et al. (2014) for Lso quantification and immunolocalization. DNA from pools of 50 guts was purified following the protocol of blood/tissue DNA extraction kit (Qiagen, Hilden, Germany); each pool was used as an individual template for quantitative real-time PCR (qPCR) analysis. Thus, each pool of 50 guts represented one replicate, there were three replicates for each combination of exposure time point and haplotype. Each replicate was obtained by using independent LsoA‐ or LsoB-infected plants as Lso inoculum.

### Quantification of Lso

The Lso 16S rDNA specific primers (LsoF: 5'-CGAGCGCTTATTTTTAATAGGAGC-3' and HLBR: 5'-GCGTTATCCCGTAGAAAAAGGTAG-3'; Li et al., 2006, 2009) were used for Lso quantification in the guts of adult psyllids, and the psyllid 28S rDNA primers (28S rDNAF: 5'-AGTTTCGTGTCGGGTGGAC -3' and 28S rDNAR: 5'-AACATCACGCCCGAAGAC-3'; Nachappa et al., 2014) were used as internal control. Quantitative PCR (qPCR) was performed using SYBR Green Supermix Kit (Bioline, Taunton, MA) according to the manufacturer’s instructions. Each reaction contained 25 ng of DNA, 250 nM of each primer, and 1X of SYBR Green Master Mix; the volume was adjusted with nuclease-free water to 10 μl. The qPCR program was 95°C for 2 min followed by 40 cycles at 95°C for 5 s and 60°C for 30 s. qPCR assays were performed using a QuantStudio™ 6 Flex Real-Time PCR System (Applied Biosystems, Foster City, CA). Reactions for all samples were performed in triplicates with a negative control in each run. In order to standardize the amount of Lso in psyllid guts, data are reported as delta Ct = (Ct of Lso gene) – (Ct of psyllid 28S gene). The biological replicates were analyzed and the average delta Ct value was used to quantify the levels of Lso. A standard curve was prepared for the quantification of Lso in the psyllid guts using a plasmid containing the Lso 16S rDNA target. For the standard curve, 10-fold serial dilutions of the plasmid were performed. The procedure for the standard curve preparation and the calculations of molecules (copies) followed published methods (Levy et al., 2011). The Lso copy number in each sample was estimated by comparing the delta Ct values of each sample to the standard curve.

### Immunolocalization of Lso in Psyllid Guts

Immunolocalization was used to visualize Lso in the Lso-exposed psyllid alimentary canal tissues. The psyllid guts were dissected in 1X phosphate-buffered saline (PBS; Sigma-Aldrich, St. Louis, MO) from adults, which were infected for 2, 3, 5, and 7 days, respectively (Figure 1A). Then the guts were fixed in 4% paraformaldehyde for 30 min at room temperature. After fixation, the guts were incubated with Sudan Black B (Sigma-Aldrich) for 20 min to quench the autofluorescence as described in Tang et al. (2019). Next, the guts were permeabilized by adding 0.1% Triton X-100 (Calbiochem/EMD Chemicals, Gibbstown, NJ) for 30 min at room temperature, and washed three times with PBS containing 0.05% Tween 20 (PBST) prior to a 1 h blocking at room temperature with blocking buffer [PBST with 1% (w/v) bovine serum albumin]. Lso immunolocalization was performed using a rabbit-derived polyclonal antibody (GenScript Corp, Piscataway, NJ) directed against Lso outer membrane protein (OMP) raised against the synthesized peptide OMP-B “VIRRELGFSEGDPIC” (Tang et al., 2019). The guts were incubated with the antibody (diluted 1: 500) overnight at 4°C. The guts were then washed three times with PBST and incubated with Alexa Fluor 594 goat anti-rabbit IgG secondary antibody (diluted 1: 2,000; Invitrogen, Carlsbad, CA) for 1 h at room temperature. The guts were washed again three times with PBST, and mounted with one drop of Vectashield mounting medium with 4',6-diamidino-2-phenylindole (DAPI; Vector Laboratories Inc., Burlingame, CA) on a microscope slide. The slide was covered with a glass coverslip and sealed with nail polish. At least 20 guts per exposure time point and haplotype were examined using an Axioimager A1 microscope (Carl Zeiss microimaging) using the rhodamine filter (594 nm, red) and the images were collected and analyzed with the Axiovision Release 4.8 software (Carl Zeiss).

### TUNEL Assay

To test the integrity of the genomic DNA in gut cells after Lso exposure, the 2-, 3-, 5-, and 7-day Lso-exposed guts were dissected as previously described. The dissected guts were fixed in 4% paraformaldehyde for 2 h at room temperature. After that, the guts were blocked by 5% bovine serum albumin in 1X PBS with 0.1% Tween 20, then incubated with TUNEL (terminal deoxynucleotidyl transferase dUTP nick end labeling) for 6 h as described in Tang et al. (2020a) and Wang et al. (2018). TUNEL staining was performed using the *In Situ* Cell Death Detection Kit (Roche, Basel, Switzerland). After washing three times in PBS, the guts were mounted using Vectashield mounting medium with DAPI as described above. At least 20 guts per exposure time point and haplotype were observed using the FITC (488 nm, green) filter. The images were collected and analyzed with Axiovision Release 4.8 software (Carl Zeiss).

We also used the apoptosis inducer Concanavalin A (ConA) by feeding, as a positive control. Following the protocol of our colleagues (Sprawka et al., 2015; Tang et al., 2020a) with modifications, the liquid diet used for psyllid feeding bioassays was prepared with a sterilized solution of 15% (w: v) sucrose and 1X PBS (Sigma-Aldrich, St. Louis, MO). ConA (MP Biomedicals, Solon, OH) was incorporated into the diet at a concentration of 2,000 μg/ml (Sprawka et al., 2015). Young adults were placed in plastic feeding chambers (*h* = 2 cm, *Φ* = 3 cm), which were covered by two sheets of Parafilm with 100 μl of the liquid diet described above in between the two layers. Next, the guts from the ConA-treated psyllids were dissected after 72 h of feeding. The dissected guts were incubated with TUNEL as described above.

### Nuclear Morphology and Actin Cytoskeleton Architecture

To investigate whether Lso impact the nuclear morphology and actin cytoskeleton architecture of the psyllid gut cells, the guts from 2-, 3-, 5-, and 7-day Lso-exposed psyllids were dissected and fixed as previously described. After fixation, the guts were first incubated with Sudan Black B to remove autofluorescence and then incubated with phalloidin (dilution 1:200; Invitrogen). The guts were washed again three times with PBST and mounted with one drop Vectashield mounting medium with DAPI as previously described. At least 20 guts per exposure time point and haplotype were examined using Axioimager A1 microscope (Carl Zeiss microimaging) using the FITC (488 nm, green) filter. The images were collected and analyzed with Axiovision Release 4.8 software (Carl Zeiss).

### Expression of Apoptosis-Related Genes

The apoptosis-related genes were identified through searching the psyllid transcriptome datasets in our previous studies (Nachappa et al., 2012a; Tang and Tamborindeguy, 2019). Three caspases and four IAPs with BIR domain(s) were selected to evaluate the gene expression in the psyllid gut upon Lso infection. The gene names and codes are listed in Supplementary Table S1. Of the three caspases, caspase-2 is the initiator caspase, and caspases 1 and 3 are effector caspases. Of the four IAPs, one DIAP1-like gene (IAP1), one DIAP2-like gene (IAP2), and two Deterin/Survivin-like genes (IAPP5 and IAPP5.2) were identified. About 7-day-old Lso-free female adult psyllids were transferred to LsoA‐ or LsoB-infected plants for 2, 3, 5, and 7 days (Figure 1A). The Lso-free colony was used as a control. Three replicates were conducted for each treatment, and each replicate had 200 psyllid individuals. After exposure, the psyllid guts were dissected under the stereomicroscope (Olympus) as previously described. RNA from pools of guts was purified using the RNeasy Mini Kit (Qiagen). Genomic DNA was eliminated by DNase I treatment with Turbo DNase (Ambion, Invitrogen). Then the total RNA was reverse transcribed using the Verso cDNA Synthesis kit (Thermo, Waltham, MA) and anchored-Oligo (dT) primers following the manufacturer’s instructions. The expression of apoptosis-related genes in the psyllid guts upon Lso infection was evaluated by qPCR using SensiFAST SYBR Hi-ROX Kit (Bioline) according to the manufacturer’s instructions. The primers for qPCR are listed in Supplementary Table S2. The qPCR reaction and program were performed as described above. The relative expression of the candidate genes were estimated with the delta CT method (Schmittgen and Livak, 2008), using two reference genes elongation factor-1a (GenBank KT185020) and ribosomal protein subunit 18 (GenBank KT279693; Ibanez and Tamborindeguy, 2016).

### RNA Interference of IAPP5.2 and Its Effects on Lso Acquisition and Transmission

The T7 promoter was incorporated into the 5'-end of the forward and reverse IAPP5.2 primers (Supplementary Table S2) to enable *in vitro* transcription. The targeted region was blasted against the transcriptome of potato psyllid (Nachappa et al., 2012a) to ensure its specificity. Double-stranded RNAs (dsRNA) were synthesized *via* the MEGAscript RNA interference (RNAi) kit (Invitrogen) using PCR-generated DNA template that contained the T7 promoter sequence at both ends. The dsRNA quality was monitored by agarose gel electrophoresis. dsRNA of the *Aequorea victoria* green fluorescent protein (GFP) was used as a control. RNAi was performed by feeding assay using a liquid diet with dsRNA. Specifically, the liquid diet used for psyllid feeding bioassay was prepared with a sterilized solution of 15% (w:v) sucrose and 1X PBS (Sigma-Aldrich, St. Louis, MO). dsRNA was incorporated into the diet at a concentration of 500 ng/μl. Young female adults from the Lso-free colonies were collected and placed in plastic feeding chambers (*h* = 2 cm, *Φ* = 3 cm) for 4 days. The chambers were covered by two sheets of parafilm with 60 μl of the above liquid diet in between the two layers. The diet was refreshed every 2 days. There were three replicates with 30 psyllid individuals each. After silencing, the gene expression of apoptosis-related genes and TUNEL assays were performed as described above.

To examine the effects of silencing IAPP5.2 on the accumulation of LsoA or LsoB in the psyllid guts, 30 young psyllids were allowed to feed on LsoA‐ or LsoB-infected tomato plants for 2 days before a 4-day feeding on the dsRNA-containing diet. The same number of guts from the control (dsGFP) and dsIAPP5.2 RNAi treatments were dissected for Lso quantification. Three replicates were conducted for the Lso accumulation assays.

To examine the effects of silencing IAPP5.2 on the transmission of LsoA or LsoB by potato psyllid, young Lso-free adult psyllids were exposed to LsoA‐ or LsoB-infected plants for a 2-day AAP as described above. Then, the Lso-infected psyllids were fed with the dsRNA-containing diet for 4 days. Groups of four treated psyllids were transferred to 10 4-week-old non-infected recipient tomato plants for an 11-day inoculation access period according to the latent period of Lso (Tang et al., 2020b). Then every 4 days, the same batch of LsoA‐ or LsoB-exposed psyllids was continuously transferred to a new set of 10 non-infected recipient tomato plants. The sequential transmissions were stopped at day 25 because of psyllid mortality. In total, three rounds of transfer were conducted. Around 4 weeks after the end of the inoculation access period, the plants were tested for Lso infection as described above. The transmission assays were performed three times.

### Data Analysis

All data were analyzed with JMP Version 12 (SAS Institute Inc., Cary, NC, United States). For gene expression results upon Lso infection, different letters indicate statistical differences at *p* < 0.05 using one-way ANOVA with Tukey’s *post hoc* test. For RNAi assays, gene expression and quantification of Lso were determined with Student’s *t*-tests. For transmission assays, the percentage of infected plants from the three replicated experiments of each time point and treatment were determined using a logistic regression model, which was fit to evaluate the effects of two factors (RNAi treatments and the days post acquisition) on the probability that a plant would become infected. The log-odds model is given by:

$$\begin{array}{l} \log\left[\frac{p}{\left(1-p\right)}\right]=b0+b1\;\mathrm{RNAi treatments}+b2\;\mathrm{Days}+\\ b3\;\mathrm{RNAi treatments}*\mathrm{Days}+e \end{array}$$

where p = probability plant becomes infected after being exposed to the Lso a given number of days after the potato psyllid was exposed to the Lso and dsRNA, and p/(1-p) is the odds of an exposed plant becoming infected.

## Results

### Accumulation of LsoA and LsoB in the Gut of Adult Potato Psyllids

Lso was quantified in the gut of psyllids following different AAPs (2-, 3-, 5-, and 7-day). The results showed that both LsoA and LsoB were detectable with ~10,285 copies in pools of 50 guts of adult potato psyllids at the beginning of infection (2-day AAP; Figure 1B). Similar Lso titers were measured after a 3-day AAP. However, after 3 days, LsoB titer increased rapidly with on average ~60,031 copies after a 5-day AAP, and reached a high level of ~98,896 copies after a 7-day AAP. In contrast, the titer of LsoA did not increase over time. In addition, LsoB titer was significantly higher than LsoA titer after 5 days of AAP (*p* < 0.05).

Comparable results were obtained by immunolocalization of Lso in the psyllid guts. No obvious LsoA or LsoB signal could be observed after a 2-day AAP (Figure 1C). However, LsoA‐ or LsoB-derived signal could first be observed after the 3 days of AAP. In accordance with the quantification analysis by qPCR, LsoA signal remained low after the 5-day and 7-day AAPs; however, increasing LsoB signal was observed after the 5-day AAP. In particular, the LsoB-derived signal was strong and more widespread after 7 days of AAP (Figure 1C).

### Lack of Evidence of Apoptosis in the Lso-Exposed Gut of Adult Potato Psyllids

**Figure 2:**
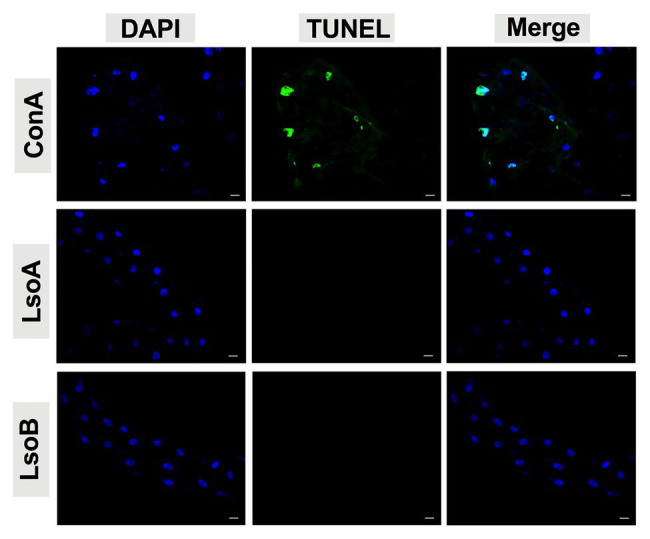
Detection of apoptosis in Lso-exposed potato psyllid gut using TUNEL. The newly Lso-exposed guts were stained using TUNEL to detect the apoptotic signals (green) and counterstained with 4',6-diamidino-2-phenylindole (DAPI) to show the nuclei (blue) of the gut cells. The ConA treatment was used as a positive control. Only 7-day LsoA‐ or LsoB-exposed guts are shown, similar results were found for the other AAPs. Scale bar is 20 μm.

**Figure 3:**
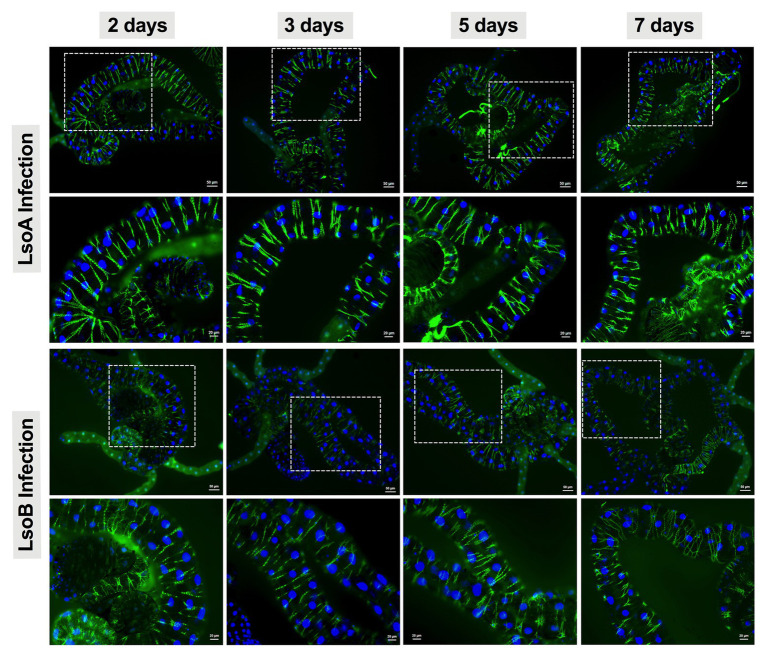
Nuclear morphology and actin cytoskeleton organization in gut cells following Lso exposure. All the guts showed uniform and round shape of cell nuclei (blue) and organized structure of the actin filaments (green). The white dashed rectangle indicates the enlargement region of upper panels. The bars of **upper and lower panels** are 50 and 20 μm, respectively.

As indicated above, Lso was detectable in the guts of adult potato psyllids from the beginning of infection (2-day AAP), then the DNA fragmentation of the psyllid gut cells was tested following the 2-, 3-, 5-, and 7-day based on TUNEL assay. However, no signal of DNA fragmentation was detected in the newly Lso-infected guts (Figure 2 shows he results following the 7-day AAP). In contrast, several cell nuclei from the ConA-treated psyllid guts (positive control) exhibited signals of DNA fragmentation (Figure 2). Also, the nuclear morphology and actin cytoskeleton architecture of the psyllid gut cells were observed following 2-, 3-, 5-, and 7-day acquisition of LsoA or LsoB based on DAPI and phalloidin staining, respectively. The results showed that both LsoA‐ or LsoB-exposed gut nuclei appeared regularly dispersed in the cells and were of uniform round shape and size based on DAPI staining (blue, Figure 3). The actin filaments in those guts appeared organized as well (green, Figure 3).

### IAPP5.2 Was Induced During the Period That Lso Translocated Into the Gut Cells

To test apoptotic responses upon Lso infection at the molecular level, three caspases and four IAPs with BIR domain(s) were selected to evaluate the gene expression in the psyllid gut at the early stage of Lso infection.

**Figure 4:**
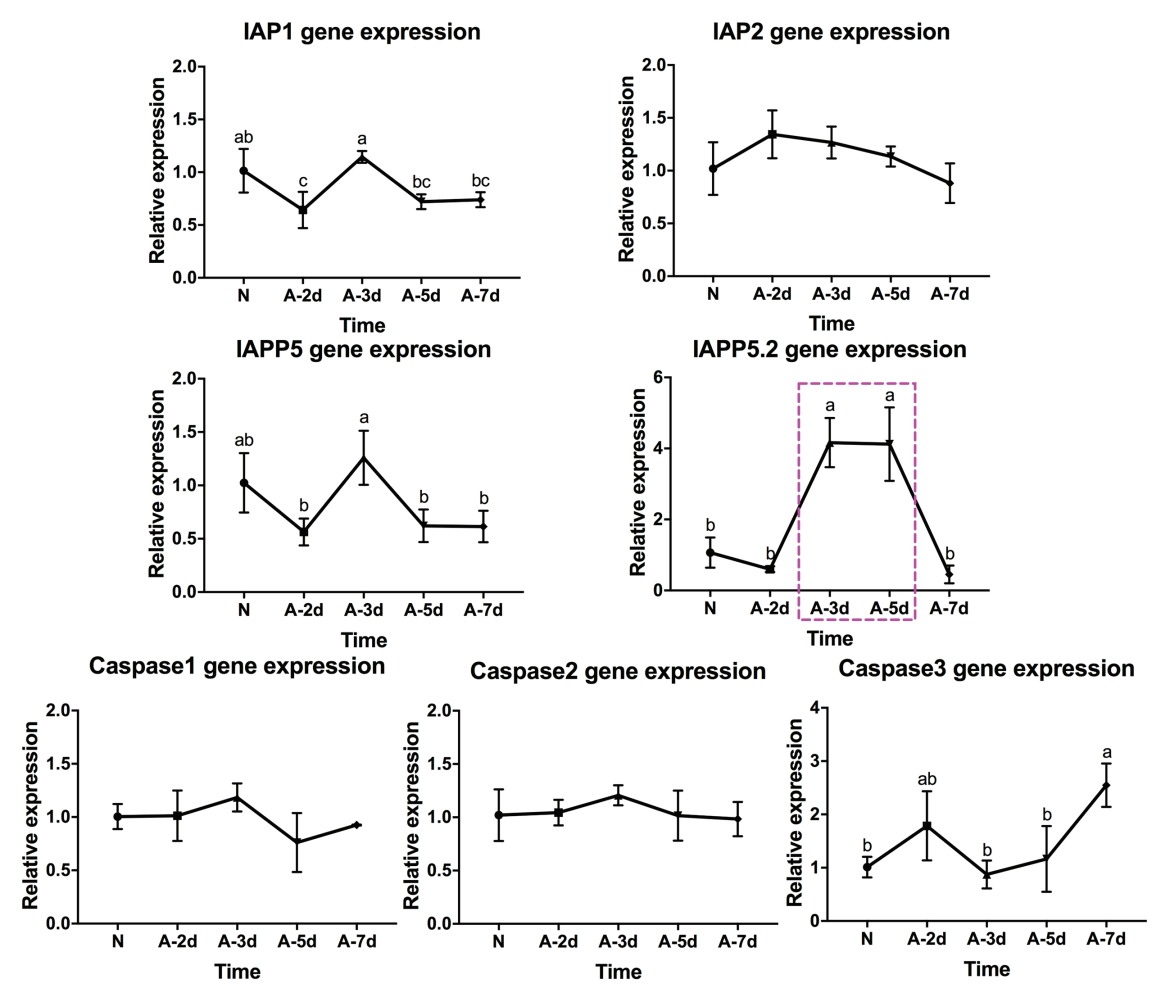
Regulation of apoptosis-related genes in the psyllid gut upon LsoA infection. Data represent means ± SD of three independent experiments. Different letters indicate statistical differences at *p* < 0.05 using one-way ANOVA with Tukey’s *post hoc* test. N: Lso-free colonies; A-2d, A-3d, A-5d, and A-7d indicate the psyllids were infected by LsoA for 2, 3, 5, and 7 days, respectively. The pink dashed rectangle indicates the significant upregulation profile of IAPP5.2 gene. The IAPP5.2 gene also had the opposite regulation profile with the effector caspase-3.

In response to LsoA acquisition, two inhibitors IAP1 and IAPP5 were upregulated at day 3 compared to days 2, 5, and 7. IAPP5.2 was significantly upregulated at days 3 and 5 compared to non-infected guts as well as after a 2‐ and 7-day AAP. Caspase-3 was significantly upregulated at day 7 compared to the uninfected guts and guts following the 3‐ and 5-day AAP. Furthermore, caspase-3 had the opposite expression profile than IAPP5.2. No significant regulation of IAP2 and the other two caspases was observed in response to LsoA acquisition (Figure 4).

**Figure 5:**
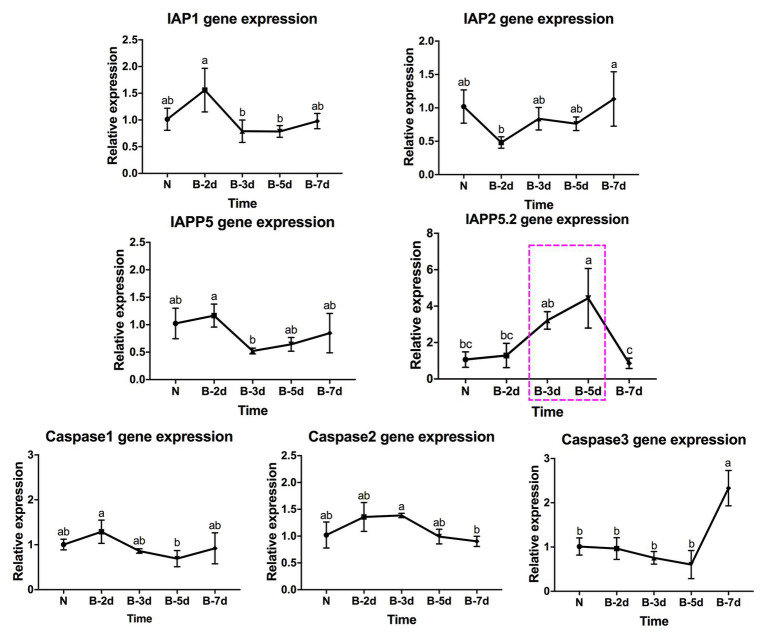
Regulation of apoptosis-related genes in the psyllid gut upon LsoB infection. Data represent means ± SD of three independent experiments. Different letters indicate statistical differences at *p* < 0.05 using one-way ANOVA with Tukey’s *post hoc* test. N: Lso-free colonies; B-2d, B-3d, B-5d, and B-7d indicate the psyllids were infected by LsoB for 2, 3, 5, and 7 days, respectively. The pink dashed rectangle indicates the significant upregulation profile of IAPP5.2 gene. The IAPP5.2 gene also had the opposite regulation profiles with the effector caspase-3.

In response to LsoB acquisition, the inhibitor IAP1 was upregulated at day 2 compared to days 3 and 5. IAP2 was significantly upregulated at day 7 compared to day 2. In addition, IAPP5 was significantly upregulated at day 2 compared to day 3. Similar to LsoA infection, IAPP5.2 was upregulated at day 3 compared to day 7, and at day 5 compared to the uninfected gut as well as to the 2‐ and 7-day AAP. Of the three caspase genes, caspase-1 was upregulated at day 2 compared to day 5 and caspase-2 was upregulated at day 3 compared to day 7 infection, however, in both cases the changes in gene expression were relatively minor. On the other hand, caspase-3 was upregulated in response to LsoB acquisition at day 7, and it had the opposite expression profile as IAPP5.2 (Figure 5).

In summary, IAPP5.2 was significantly upregulated in response to both LsoA and LsoB between the 3‐ and 5-day AAP, which is the period that Lso translocated into the gut cells as indicated by immunolocalization of Lso in the psyllid guts.

### Silencing of IAPP5.2 Induces Apoptosis in the Gut of Adult Potato Psyllids

**Figure 6:**
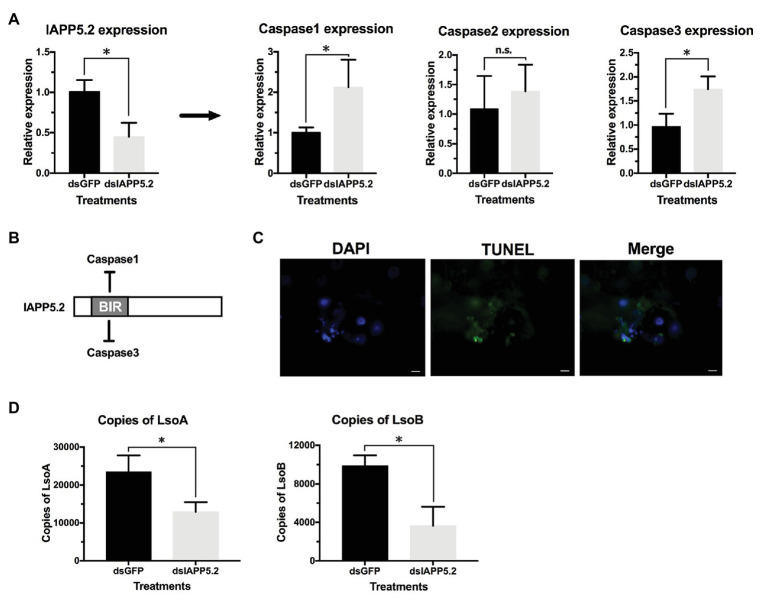
Silencing of IAPP5.2 and its effect on Lso acquisition in psyllid gut. **(A)** Relative gene expression of IAPP5.2, caspases-1, 2, and 3 in psyllid gut following RNA interference (RNAi) silencing of IAPP5.2. **(B)** IAPP5.2 probably binds and interacts with caspases 1 and 3 *via* the baculoviral IAP repeat (BIR) domain. **(C)** DNA fragmentation of guts could be observed based on TUNEL assays after silencing of IAPP5.2. **(D)** Quantification of LsoA and LsoB in the potato psyllid gut after silencing of IAPP5.2. Silencing of IAPP5.2 in the guts of psyllids resulted in reduced LsoA and LsoB accumulation. * indicates *p* < 0.05.

Because IAPP5.2 had the opposite expression profile as the effector caspase-3 in response to both LsoA and LsoB, we hypothesized that IAPP5.2 could be the key inhibitor for caspase activity inhibition in the apoptosis pathway of potato psyllid. Then we silenced IAPP5.2 gene expression by RNAi and evaluated the expression of caspases. The oral delivery of dsRNA resulted in 55% decrease of the expression of IAPP5.2 in the gut (Figure 6A), and in the upregulation of the transcriptional expression of the two effector caspases, caspase-1 and caspase-3. However, it had no impact on the initiator caspase, caspase-2 (Figure 6A). Therefore, IAPP5.2 could bind and interact specifically with the two effector caspases with its BIR domain (Figure 6B; Supplementary Figure S1). Importantly, the upregulation of two effector caspases was accompanied by DNA fragmentation in the IAPP5.2-silenced guts based on TUNEL assay (Figure 6C).

### Silencing of IAPP5.2 Decreases Lso Acquisition and Transmission Efficiency

**Figure 7:**
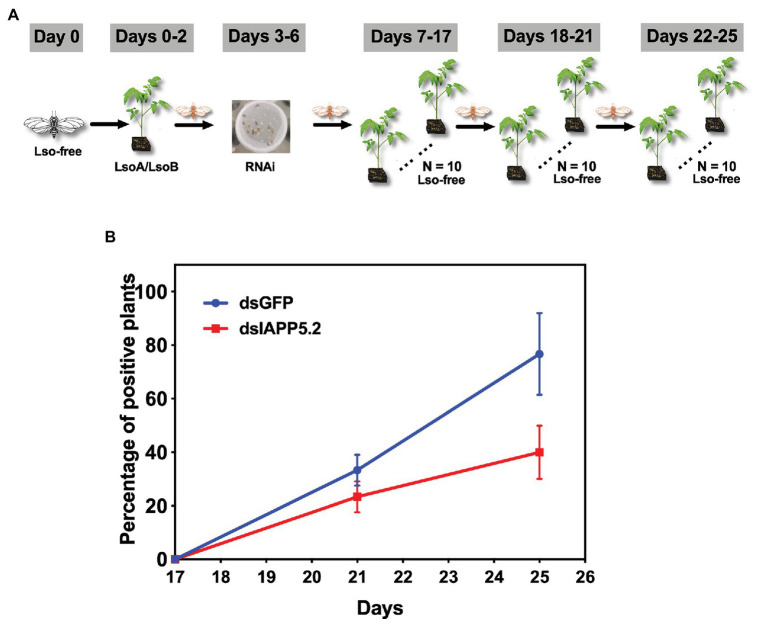
The effect of silencing of IAPP5.2 on LsoB transmission by potato psyllid. **(A)** Adult psyllids were given a 2-day AAP on Lso infected tomato plants and fed with the double-stranded RNAs (dsRNA)-containing diet for 4 days. Then, groups of five adult psyllids were transferred to 10 recipient non-infected tomato plants for an 11-day inoculation access period, and each group was sequentially transferred to a new uninfected recipient plant every 4 days as shown in the schematic representation. The days showed in the figure indicate the days post acquisition. Day 0 is the initial day Lso-free psyllids were exposed to Lso-infected plants. **(B)** Silencing of IAPP5.2 decreases LsoB transmission efficiency. Data represent means ± SD of three independent experiments.

Because IAPP5.2 (survivin-like) gene was significantly upregulated during the period Lso translocated into the gut cells, we hypothesized that IAPP5.2 could be involved in the acquisition or even transmission process of Lso. We first silenced IAPP5.2 gene by RNAi and quantified Lso in psyllid gut. Interestingly, silencing of IAPP5.2 significantly decreased both LsoA and LsoB titers in the guts of adult psyllids after 6 days with a 2-day AAP (Figure 6D). Therefore, we further hypothesized that the transmission efficiency of Lso by potato psyllid were also affected by dsIAPP5.2-induced apoptosis in psyllid gut. Then we silenced the IAPP5.2 gene and performed sequential inoculation of tomato plants following a 2-day AAP. The results showed that all the recipient tomato plants were tested LsoB-negative at day 17 post acquisition, and similar LsoB transmission rates (23.3–33.3%) were observed at day 21 post acquisition between silencing of GFP (control) and IAPP5.2. However, the LsoB transmission rates were significantly decreased after silencing of IAPP5.2 (average rates = 40%) compared to the control (average rates = 76.7%), at 25 days post acquisition (Figure 7). Based on the logistic regression model, there was significance of the effects of two factors (RNAi treatments and the days post acquisition) on the probability that a plant would become infected (*p* < 0.01). However, no significance of the RNAi treatments by days interaction was observed (*p* = 0.586). Importantly, by silencing of IAPP5.2, the plants also had significantly lower odds (probability of infection) of infection at 25 days post acquisition relative to the control (silencing of GFP). For transmission assay of LsoA, none of the plants were infected by LsoA at 17, 21, or 25 days post acquisition, although the inoculated insects were tested positive.

## Discussion

A variety of pathogenic organisms can infect insects, including bacteria, viruses, and other organisms. Understanding the characteristics of pathogens and the transmission mechanisms by insect vectors will greatly contribute to create new approaches for controlling diseases caused by insect-borne plant pathogens. The innate immunity plays a critical part in the outcome of pathogens infection of insects (Kingsolver et al., 2013; Cao et al., 2015; Hillyer, 2016). Meanwhile, pathogens can exploit their host’s cell machinery and avoid the host’s immune defenses for successful replication and transmission (Vyas et al., 2015).

Apoptosis, as an important part of the innate immunity, plays a crucial role in defending against pathogens and limiting the spread of infections. In turn, many intracellular pathogens target caspase signaling as a means to impede apoptosis of the infected host cells. Different strategies are utilized by pathogens to repress the host apoptotic response *via* caspase signaling. For instance, the malaria parasite, *Plasmodium falciparum* alters the cell death pathway of the invaded mosquito midgut cells by disrupting c-Jun N-terminal kinase (JNK) signaling, which regulates the activation of the effector caspase-S2 (Ramphul et al., 2015). Another example is the baculovirus caspase inhibitors, which block apoptosis downstream of effector caspase DrICE in *Drosophila* cells (Lannan et al., 2007). In fact, inhibitors of apoptosis were discovered in baculoviruses and the BIR domain present in the IAP proteins stands for Baculovirus IAP Repeat (Crook et al., 1993). Apart from parasites and viruses, bacteria also have evolved a variety of strategies to block apoptosis at different stages within the apoptotic pathways. For example, *Chlamydia* presumably secretes proteins that result in blocking the release of cytochrome c from the mitochondria and in the inhibition of the activation of effector caspase-3 (Fan et al., 1998).

Numerous studies have focused on the immune responses of host plants to pathogens or the manipulation of plant immunity by pathogens (Dodds and Rathjen, 2010; Weiberg et al., 2013; Asai and Shirasu, 2015; Hacquard et al., 2017). Recently, several studies investigated the immune interactions between insect vectors and plant pathogens, and they found that plant pathogens, in particular plant viruses can induce programmed cell death in their vectors. The induced cell death may fill different roles: it could be a defensive mechanism to protect the insect against the invasion of the pathogens (Wang et al., 2016), or could help the pathogens be acquired or escape specific tissues (Huang et al., 2015; Chen et al., 2017). In both scenarios, these cell death responses can affect the pathogen acquisition or transmission. However, our understanding of apoptosis induction or inhibition by vector-borne plant pathogenic bacteria remains largely limited.

In our previous study, no evidence of apoptosis was observed in the gut of adult potato psyllids in response to either Lso haplotype in the case of a persistent infection (Tang and Tamborindeguy, 2019). In the present study, we focused on the potential role of apoptosis as a response in the gut of adult potato psyllids at the early stage of Lso infection. We evaluated if an apoptotic immune response in the psyllid gut occurred between the 2‐ and 7-day Lso AAP. However, the fluorescent imaging of the TUNEL assay, the nuclear morphology and the actin cytoskeleton architecture also indicated no evidence of Lso-induced abnormalities in newly Lso-exposed guts. Because pathogens can manipulate the apoptotic response of their host, we further evaluated the expression of apoptosis-related genes during this infection period. Interestingly, IAP1 and IAPP5 had similar expression profiles in response to LsoA or LsoB, and therefore these two inhibitors might be co-regulated in the psyllid gut. Furthermore, analysis of the expression of apoptosis-related genes revealed that the inhibitor of apoptosis IAPP5.2 (survivin-like) was significantly upregulated in response to both LsoA and LsoB after a 3‐ and 5-day AAP. We also noted that the effector caspase-3 had the opposite regulation profiles with IAPP5.2 in response to both LsoA and LsoB. It is possible that LsoA and LsoB suppress caspase activation and further repress the apoptotic response during early infection *via* the upregulation of IAPP5.2 (survivin-like). Indeed, survivin has been shown to prevent the host cellular apoptosis in other infection models. For instance, viral pathogens such as hepatitis B virus (HBV) and human neurotropic virus JC virus have been shown to suppress host caspase activation and apoptosis in a survivin-dependent manner (Marusawa et al., 2003; Pina-Oviedo et al., 2007). In addition to viruses, during the infection by the intracellular parasite *Cryptosporidium parvum* mRNA levels of survivin increases between 12 and 48 h post infection in gut cells. Moreover, siRNA depletion of survivin significantly increases the effector caspase-3/7 activity and further reduces the parasite growth (Liu et al., 2008). siRNA depletion also suggested the role of survivin in blocking apoptosis especially more critical after 24 h of *C. parvum* infection. In the present study, we also found that silencing of IAPP5.2 significantly upregulated the transcriptional expression of the effector caspases 1 and 3 and resulted in the occurrence of TUNEL-positive cells. Therefore, IAPP5.2 could inhibit these caspases and the apoptotic response in the psyllid gut cells. This study represents the first report showing that bacterial pathogen might repress host caspase activation and apoptosis in a survivin-dependent manner.

In humans, the apoptosis inhibitors XIAP, c-IAP1, and c-IAP2 are able to directly inhibit caspase-3, -7, and -9, while survivin binds specifically to the effector caspase-3 and -7 rather than the initiator caspases (Shin et al., 2001; Liu et al., 2008). Very little is known about apoptosis in phloem-feeding insects or the role of different proteins involved in this process. Based on our phylogenetic study, caspase-2 is the initiator caspase, while caspases 1 and 3 are effector caspases (Tang and Tamborindeguy, 2019). Thus, in psyllids also, the survivin-like gene could regulate the effector caspases rather than initiator caspase. We noted that at 7-day infection, the caspase-3 gene was upregulated; however, we could not observe apoptosis in the psyllid gut. This may reflect the existence of a potato psyllid response to Lso since Lso, in particular LsoB, is detrimental to the vector fitness (Nachappa et al., 2012b; Yao et al., 2016; Frias et al., 2020). Although caspase-3 was upregulated at day 7 of the infection, it might not have reached or exceeded the threshold to trigger an intracellular apoptotic immune reaction (Maiuri et al., 2007). Another possibility is that apoptosis induction in potato psyllid is regulated by the two effector caspases (caspases 1 and 3). The fact is that Lso still successfully crosses the gut cells, therefore, Lso seems to be preemptive, and has evolved to repress or reduce the apoptotic response in the psyllid gut. This mechanism may help Lso successfully translocate and colonize into the gut cells, and further affect its transmission efficiency.

**Figure 8:**
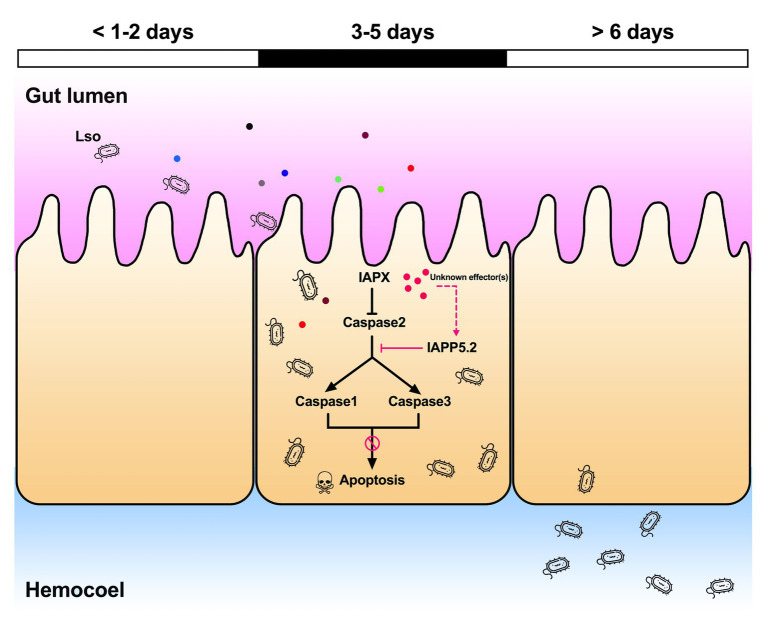
Model of the Lso-repressed apoptotic response in the psyllid gut during early infection. At days 3 and 5 of the infection when Lso first translocate into gut cells, Lso attenuate the psyllid immune responses by upregulating the inhibitor of apoptosis IAPP5.2 gene *via* unknown bacterial effectors (pink dots). The upregulation of IAPP5.2 specifically blocks the activation of effector caspases 1 and 3, which further inhibits the apoptotic response in potato psyllid gut. Other colored dots represent different Lso effectors.

Indeed, the depletion of IAPP5.2 significantly decreased LsoA and LsoB accumulation in the psyllid gut. Lso ingestion occurred before dsRNA ingestion, therefore any change in Lso accumulation was not linked to a potential behavioral change due to silencing or induced apoptosis, such as a reduction in feeding which could affect Lso ingestion. Therefore, these results indicate that the accumulation of Lso in the psyllid gut could require survivin expression within the infected host cells. Unexpectedly, LsoA titer was higher in the control and silenced insects than LsoB, which is contrary to the results obtained in the accumulation assays. This difference could arise from the use of different donor plants, even if the titer in those plants was within the range commonly measured in LsoA-infected plants (Herrera et al., 2018). The only difference in the experimental set-up between these two experiments is that insects were allowed to feed on the dsRNA solution after exposure to the infected plants. Whether this could affect Lso titer in the gut needs to be investigated. In addition, we demonstrated that the LsoB transmission rates by adult potato psyllid were significantly decreased after silencing of IAPP5.2 at 25 days post acquisition. As summarized in Figure 8, it seems that psyllids start to feed plant phloem within 2 days, and around day 3, Lso enter the gut cells and are replicating. Indeed, Lso-derived signal could be observed after the 3 days of AAP. However, Lso meet the immune response of the host gut cells. Lso seem to be preemptive and could induce the inhibitor of apoptosis IAPP5.2 at the early stage of infection. The resulting attenuation of the apoptotic response in the psyllid gut favors Lso, which can replicate safely in the gut cells. This is probably one of the strategies Lso utilizes to successfully colonize and further facilitate its transmission within the potato psyllid at the gut interface. But how does Lso regulate the expression of IAPP5.2 is still unknown, however, bacterial effector proteins could be involved (Levy et al., 2019).

Both the studies on the Asian citrus psyllid-*C*Las system and the present study suggested that apoptosis could affect the bacterial pathogen acquisition and transmission by insect vectors. As indicated above, the citrus Huanglongbing pathogen, *C*Las, was reported to induce apoptosis in the gut of the adult vector of the Asian citrus psyllid while there was no evidence of apoptosis in the nymphal gut (Ghanim et al., 2016; Mann et al., 2018) and it was suggested that this apoptotic response in adults might contribute to their reduced efficiency to acquire and transmit *C*Las compared to nymphs (Inoue et al., 2009; Ghanim et al., 2016). In addition, no evidence of apoptosis was observed in the gut of adult potato psyllids in response to either Lso haplotype in the case of a persistent infection (Tang and Tamborindeguy, 2019) or upon infection in this study, which is also consistent with the efficient transmission of each Lso haplotype by adults that acquired the pathogen during the nymphal stage. Therefore, an apoptotic immune response seems to be a key factor in *Liberibacter* bacteria transmission by psyllids. In fact, psyllids and another hemipteran pest such as aphids have “odd” immune systems likely lacking common immune pathways and antimicrobial effectors (Gerardo et al., 2010; Arp et al., 2016). For instance, the genome of pea aphid *Acyrthosiphon pisum* has been shown to lack genes in the immune deficiency (IMD) pathway (Gerardo et al., 2010), and the IMD pathway gene transcripts were also not found in the transcriptome of the Asian citrus psyllid and potato psyllid (Nachappa et al., 2012a; Kruse et al., 2017).

Quantification of each Lso haplotype following early exposure times to infected plants revealed that LsoA and LsoB population increased with different rates in the psyllid guts. LsoB density increased rapidly after 3 days, while the increase in LsoA copy number was much slower. During the period of early infection, LsoB has probably entered the gut cells and is replicating, however, LsoA is not actively replicating yet. Our previous studies determined that this delay in LsoA accumulation in the psyllid gut is accompanied by a significant decrease in Lso transmission (Tang et al., 2020b). This probably could explain why none of the plants tested positive for LsoA infection in the transmission assays. In addition, the differences of LsoA and LsoB titer in the gut of adult psyllids could be the result of differences of bacterial pathogenicity or the elicited psyllid immune responses. Indeed, differences in virulence between these two haplotypes were determined in association with their host plants and insect vector: in both cases, LsoB was found to be more pathogenic (Yao et al., 2016; Herrera et al., 2018; Harrison et al., 2019). Similarly, the vector immune response could be one factor explaining the different Lso accumulation profiles. It appears that similar to LsoB, LsoA might stop the apoptotic response in the psyllid gut, as showed in this study; however, other immune responses might be differentially elicited in response to each Lso haplotype. It is therefore possible that LsoB is able to better defend itself against the psyllid immunity. As a consequence, LsoB could be acquired with higher efficiency than LsoA.

In summary, our study demonstrates for the first time that the regulation of apoptosis and the suppression of caspase activation observed in the Lso-exposed cells were due to the upregulation of survivin and was probably mediated by the effector caspases 1 and 3 with unknown bacterial effector (s). This is not only the first study showing that bacterial pathogen represses the host caspase activation and apoptosis in a survivin-dependent manner, but it is the first report indicating that a plant bacterial pathogen can impede the immunity in its insect vector.

## Data Availability Statement

The original contributions presented in the study are included in the article/Supplementary Material, further inquiries can be directed to the corresponding author.

## Author Contributions

CT and X-TT designed the study, performed the data analyses, and wrote the manuscript. X-TT, KF, and AM were responsible for performing the experiment. All authors contributed to the article and approved the submitted version.

### Conflict of Interest

The authors declare that the research was conducted in the absence of any commercial or financial relationships that could be construed as a potential conflict of interest.
