# Supplementary material for: Liberibacter, A Preemptive Bacterium: Apoptotic Response Repression in the Host Gut at the Early Infection to Facilitate Its Acquisition and Transmission
Source: Front Microbiol. 2020 Dec 23;11:589509. doi: 10.3389/fmicb.2020.589509 (PMC7786102; doi:10.3389/fmicb.2020.589509)
Supplement: Supplementary file 1 [file Data_Sheet_1.docx]

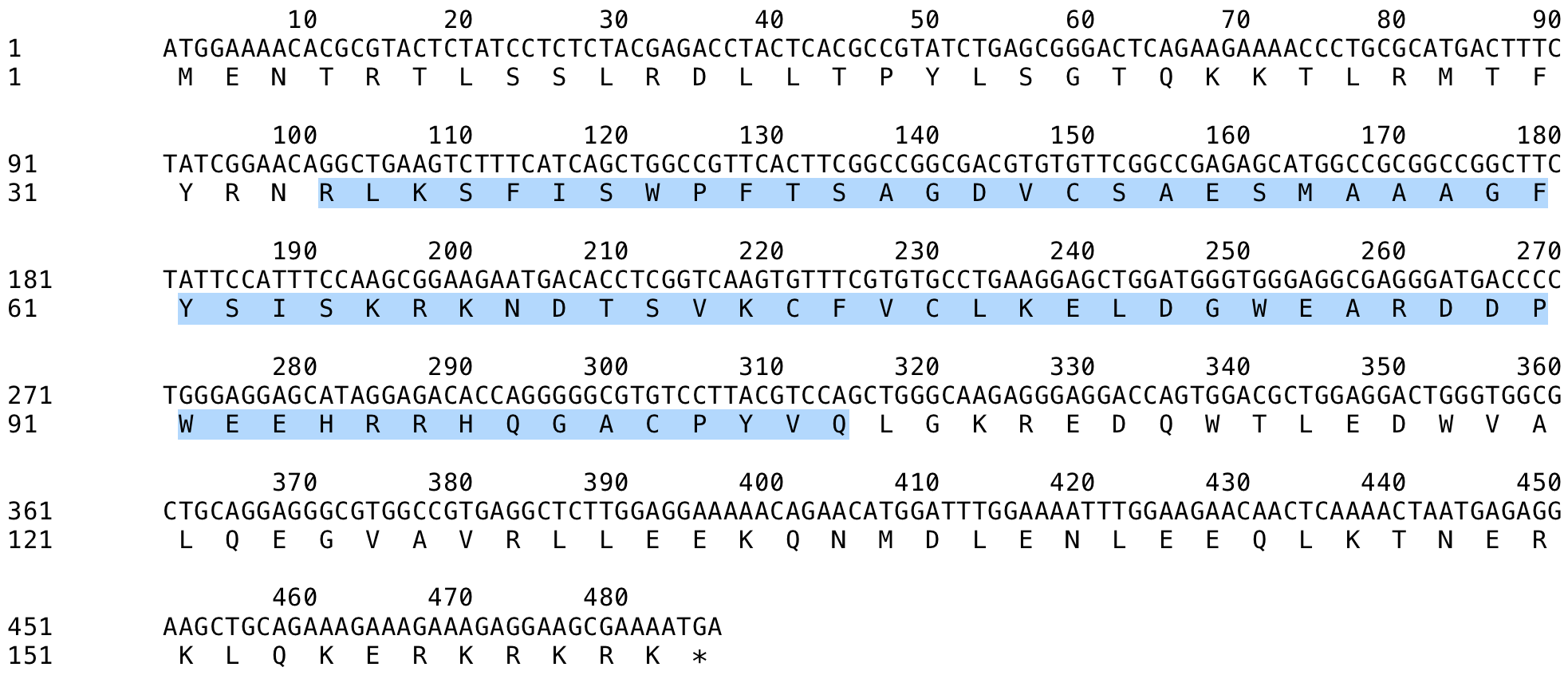


**Figure S1**. DNA and protein sequence of IAPP5.2 gene. The highlight indicates the Baculovirus IAP Repeat (BIR) domain.
