# Supplementary material for: Liberibacter, A Preemptive Bacterium: Apoptotic Response Repression in the Host Gut at the Early Infection to Facilitate Its Acquisition and Transmission
Source: Front Microbiol. 2020 Dec 23;11:589509. doi: 10.3389/fmicb.2020.589509 (PMC7786102; doi:10.3389/fmicb.2020.589509)
Supplement: Supplementary file 2 [file Data_Sheet_2.docx]

**Table S1. The gene name and code of apoptosis-related genes.**

| **Gene name** | **Code** |
| --- | --- |
| Inhibitor of apoptosis isoform X1 | IAP1 |
| Baculoviral IAP repeat-containing protein 2 | IAP2 |
| Baculoviral IAP repeat-containing protein 5 | IAPP5 |
| Baculoviral IAP repeat-containing protein 5.2-like isoform X1 | IAPP5.2 |
| Caspase-1 isoform X1 | Caspase1 |
| Caspase-2-like | Caspase2 |
| Caspase-3 (CASP3) | Caspase3 |

**Table S2. The qPCR and RNAi primers used in this study.**

| **Gene Code** | **qPCR primers (5'-3')** |
| --- | --- |
| IAP1 | F: AACAAGTACAGCGGTGACGA |
|  | R: TTTACACAGGCGACCATCAG |
| IAP2 | F: AGCAGAACACAGGAGGCAAA |
|  | R: TGATTAGGCAGTCCACCATTC |
| IAPP5 | F: AAAAGGGTTCTTGCACACCA |
|  | R: CAGAATATGCACTTGGCACA |
| IAPP5.2 | F: GACCTACTCACGCCGTATCTG |
|  | R: CTTCAGCCTGTTCCGATAGAA |
| Caspase1 | F: CAAGGAGATGGTCTGGATGG |
|  | R: ATGAGGAAGTCAGCGTGGAG |
| Caspase2 | F: ATGTCCCCAGCAATGGTATC |
|  | R: CACAGGGTGTTGACTTCTTCA |
| Caspase3 | F: AAGCTGGATGGTGGAGTACG |
|  | R: CAGCATAGGAGGGGATCTTG |
| **RNAi** | **PCR primers (5'-3')** |
| dsIAPP5.2 | F*: *TAATACGACTCACTATAGGGAGA*GCTTCTATTCCATTTCCAAG |
|  | R*: *TAATACGACTCACTATAGGGAGA*CGCTTCCTCTTTCTTTCTTT |

*The italic sequences are T7 promoter sequences.
